# Oxidative Stress and Its Clinical Applications in Dementia

**DOI:** 10.1155/2013/319898

**Published:** 2012-08-30

**Authors:** Peizhong Mao

**Affiliations:** ^1^The Division of Neuroscience, Oregon National Primate Research Center, Oregon Health & Science University, 505 NW 185th Avenue, Beaverton, OR 97006, USA; ^2^The Departments of Physiology and Pharmacology, Public Health and Preventive Medicine, Oregon Health & Science University, Portland, OR 97239, USA

## Abstract

Dementia is a complex disorder that mostly affects the elderly and represents a significant and growing public health burden in the world. Alzheimer's disease (AD)- associated dementia and dementia with Lewy bodies (DLB) are the most common forms of dementia, in which oxidative stress is significantly involved. Oxidative stress mechanisms may have clinical applications, that is, providing information for potential biomarkers. Thus brain-rich peptides with an antioxidant property, such as CART (cocaine- and amphetamine-regulated transcript), may be promising new markers. This paper summarizes the progress in research regarding oxidative stress in dementia with a focus on potential biomarkers in the cerebrospinal fluid (CSF) in the main forms of dementia. Other central and peripheral biomarkers, especially those considered oxidative stress related, are also discussed. This paper aims to provide information to improve current understanding of the pathogenesis and progression of dementia. It also offers insight into the differential diagnosis of AD and DLB.

## 1. Introduction

Dementia is a multisystem-related neurodegenerative disorder. According to the DSM-IIIR (*the Diagnostic and Statistical Manual*, 3rd edition, revised) the essential feature of dementia is impairment in short- and long-term memory, associated with impairment in abstract thinking, impaired judgment, other disturbances of higher cortical function, or personality change. The disturbance is severe enough to interfere significantly with work or usual social activities or relationships with others. The diagnosis of dementia is not made if these symptoms occur in delirium. The DSM-IIIR definition of dementia is reliable and is routinely used in clinical guidelines [1, 2].

There are several forms of dementia, including dementia associated with Alzheimer's disease (AD), dementia with Lewy bodies (DLB), corticobasal degeneration/dementia (CBD), frontotemporal dementias (FTD) (also known as frontotemporal lobar degenerations or FTLD), vascular dementia (VAD), and prion diseases such as Creutzfeldt-Jakob Disease (CJD) [1, 3]. Among all forms of dementia, Alzheimer's dementia and dementia with Lewy bodies are the most common. 

AD is accounting for 60–80% of the total number of dementia, characterized by extracellular fibrillar amyloid *β* (A*β*), especially long form 42 amino acids of A*β* (A*β*42) deposits (amyloid plaques), intracellular neurofibrillary tangles (NFT, phosphate-tau related), and neuronal as well as axonal degeneration in the brain [4–6].

Dementia with Lewy bodies (DLB), accounting for 15–30% of the total number of recorded cases of dementia, is the second most common form of dementia and is characterized by a dysexecutive-visuoperceptual dementia frequently accompanied by visual hallucinations, fluctuating attention, and Parkinsonism. Strategic *α*-synuclein (*α*Syn) aggregates, neuronal loss, and variable degrees of amyloid deposition constitute the key pathological features [1, 7–9]. 

Importantly, mitochondria (mt), the powerhouse in most cells, are pivotal in controlling cell survival and death. Cumulative oxidative stress, disrupted mitochondrial respiration, and mitochondrial damage are involved in various neurodegenerative disorders, including AD and DLB [4–6]. The neuropeptide CART is expressed in the nervous system and circulatory system with multiple functions. We recently found that CART is a mitochondrial booster and a novel endogenous antioxidant [10, 11], indicating its new utility in neurodegenerative diseases. For example, CART may be a disease biomarker and/or therapeutic target. 

Increasing evidence indicates the pathogenic role of oxidative stress in the early phase of neurodegeneration. As such, oxidative stress mechanisms may suggest novel disease markers. In this paper, I attempt to summarize the role of oxidative stress in dementia, with a focus on the cerebrospinal fluid (CSF) biomarkers in AD and DLB, including their relationship with CART peptide.

## 2. Oxidative Stress in the Etiopathology of Dementia

### 2.1. Oxidative Stress in Alzheimer's Dementia

A growing body of evidence suggests that AD is a multifactorial and the most common, neurodegenerative disease. Senile plaques (A*β* plaques) and neurofibrillary tangles (tau pathology) are the hallmarks in its pathology. AD is a progressive disease and AD-related pathophysiological changes including oxidative damage can occur many years and even decades before the appearance of clinical dementia syndrome. Therefore, the latest criteria proposed last year for the neuropathologic assessment of AD differ from the traditional criteria [2]. These criteria describe three defined stages in a clinical continuum that includes preclinical, mild cognitive impairment, and dementia [2].

Recent progress in AD, especially A*β*, mitochondria, and oxidative stress-related pathology has been reviewed [6, 12, 13]. Briefly, A*β* (especially A*β*42) can damage neurons by directly inducing production of reactive oxygen species (ROS), indicating A*β* peptides are probably oxidant peptides. ROS are mainly generated in mitochondria, in particular from mitochondrial dysfunction. In other words, mitochondria may have an etiological role in the development of AD. Excessive ROS damage the components of the cell, including mitochondrial DNA (mtDNA), nuclear DNA, RNA, proteins, and lipids and eventually result in synapse damage and neuronal cell death. When this occurs in the memory center, the hippocampus, and associated key brain areas, it produces the clinical manifestations of AD. 

Importantly, oxidative damage may possibly be the earliest event in AD, based on postmortem studies showing that increases in neuronal 8OHdG (8-hydroxy-2′-deoxyguanosine) and 3-nitrotyrosine, which are markers of DNA and protein oxidation, respectively, precede A*β* plaques and NFTs [6, 14, 15]. There is approximately a twofold increase in DNA damage in lymphocytes of patients with mild cognitive impairment (MCI; a strong risk for developing AD) and AD [6, 16]. Using a redox proteomics approach, several specific targets of protein oxidation such as peptidyl prolyl cis-trans isomerase and ubiquitin carboxyl terminal hydrolase 1 have been identified in the hippocampus but not in the cerebellum in AD [17], indicating that oxidatively modified proteins were consistent with biochemical or pathological alterations in AD. Furthermore, some peripheral markers of oxidative stress such as high malonaldehyde (MDA) levels (representing lipid peroxidation), low glutathione levels, and low glutathione reductase appear in MCI with a similar pattern to that observed in AD. Taken together, these findings suggest that oxidative stress may represent a signal of AD pathology, even an early signal in very mild cognitive disorders. Therefore, AD and MCI are biochemically equivalent [18–20]. Not surprisingly, increased protein and lipid oxidative damage was also found in AD brain tissue and mitochondria isolated from lymphocytes of AD patients, further indicating the role of oxidative stress in AD etiopathology [21, 22]. 

Toxic soluble A*β* oligomers appear to have synaptic receptors colocalizing with PSD-95 (postsynaptic density protein 95), and A*β*42 accumulates in dendrites in AD patients where it may cause oxidative damage, caspase activation, and finally apoptosis [23]. Docosahexaenoic acid (DHA), an essential omega-3 polyunsaturated fatty acid (PUFA), is a major component of neuronal membrane phospholipids. It is enriched in synapses and central to postsynaptic signaling and neuroprotection. It has been shown that DHA serum level is reduced in AD patients [24]. Further, the neuronal membranes of AD patients have been found to be deficient in DHA [25, 26]. The loss of DHA in AD may reflect its propensity for free radical-mediated lipid peroxidation, resulting in its conversion to neuroprostanes (F4-isoprostanes), which are elevated in AD [27, 28]. F2-isoprostanes (F2-IsoPs) are other indicators of lipid peroxidation, which are exclusive products of free radical-mediated damage to arachidonic acid and released into extracellular fluid. F(2)-IsoPs are increased in diseased regions of the brain from definite AD patients compared to controls [29]. On the other hand, a diet enriched with the omega-3 fatty acid DHA reduces amyloid burden in Alzheimer's mouse models [30, 31]. 

In addition, aging is thought to be the single most important risk factor for AD, and aging invariably decreases sensory stimuli and impacts on the thalamocortical system and its connectivity to key regions of the brain. Memory dysfunction in senescence and early AD, a function of acetylcholine decrease, is accompanied by dysfunctional basal forebrain, parietal, prefrontal, and entorhinal cortices, and indeed the hippocampus [32–34]. Cholinergic neurotransmission protects neurons from A*β* production and its toxicity, while cholinergic depletion enhances both [34]. Increasing data indicate that memory disturbances in normal elderly and early AD patients are intimately related to hypoxia, a reduction in blood supply, and glucose hypometabolism in the hippocampus and a number of key brain areas. The recent hypothesis on memory impairment in the elderly and Alzheimer's dementia, therefore, underscores age-related sensory losses, functional disconnection between strategic brain regions in conjunction with hypoxemia, and hypometabolism [34]. Notably, the hypoxia condition can increase A*β* production via the limiting enzyme *β*-site APP-cleaving enzyme 1 (BACE1) and gamma secretase. Hypoxia also stimulates ROS production, especially the radicals produced from mitochondrial complex II [6, 35, 36]. Very recently the first direct evidence in living humans that ischemia acutely increases A*β*42 levels has been reported [37].

Further, decreased cerebral microperfusion may be partially due to altered metabolism of nitric oxide (NO), which mediates the effect of endothelium-derived relaxing factor in blood vessels and plays a part in neuronal communication in the brain. The NO synthase (NOS) is expressed in the brain and regulated by many different factors [38, 39]. Human brain microvascular endothelial cell experiments showed that inhibition of endothelial NOS (eNOS) with the specific NOS inhibitor L-NAME (N(G)-nitro-l-arginine methyl ester) led to increased APP and BACE1 protein levels, as well as increased secretion of A*β*42 (*P* < 0.001). Animal experiments showed brain tissue from eNOS (−/−) mice had statistically higher APP and BACE1 protein levels, as well as increased BACE1 enzyme activity and A*β* level [40]. These data suggest that endothelial NO plays an important role in modulating APP expression and processing within the brain and cerebrovasculature and that a possible molecular link between cardiovascular risk factors and AD identified as endothelial dysfunction is specifically decreased bioavailability of nitric oxide. 

Interestingly, asymmetrical dimethylarginine (ADMA) is an endogenous inhibitor of NOS and may alter NO production during pathological conditions. It has been found that ADMA plasma concentrations were increased, while CSF ADMA concentrations were decreased in AD patients, and there was a significant association between decreasing CSF ADMA levels and the severity of cognitive impairment [41]. Decreased levels of CSF ADMA might lead to a cerebral increase of NO, peroxynitrite production, and oxidative protein damage, while elevated ADMA in plasma might be a contributing factor for AD through alterations of NO metabolism and decreased cerebral microperfusion. 

Maintaining iron homeostasis is a critical issue for normal cell and brain functions since iron is an essential component of proteins containing iron-sulfur clusters, heme, and diiron-oxo metal centers that are involved in important cellular processes like oxygen transport, mitochondrial respiration, or DNA synthesis. Iron dyshomeostasis may be a causative factor for AD and other neurodegenerative disorders [42–44]. In living organisms, iron can be found in its reduced ferrous (Fe^2+^) and its oxidized ferric (Fe^3+^) states. Severe iron deficiency will cause growth arrest and cell death, while excess free iron is involved in the Fenton redox reaction, which catalyzes the conversion of ROS to the highly reactive hydroxyl radical; further APP expression is regulated by iron [43, 45]. Iron accumulation was observed in the brain regions (such as hippocampus) that are affected by A*β* deposition and neurons that contain neurofibrillary tangles in AD patients [43]. Recently, increased iron and free radicals have been found in both the cortex and the cerebellum of preclinical AD and MCI cases [46].

**Figure 1:**
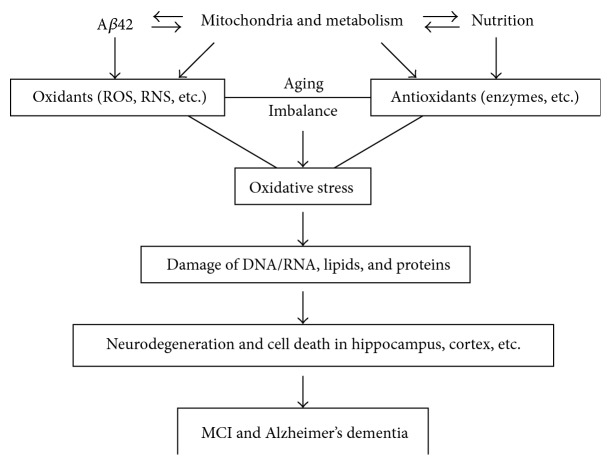
Oxidative stress in MCI and Alzheimer's dementia.

Overall, in the majority of AD, dysfunction in mitochondria and metabolism produces excessive free radicals (ROS/RNS) and A*β* that damage essential cellular components such as nucleic acids (DNA/RNA), lipids, and proteins in the brain; oxidative damage is one of the earliest events in the development of AD, including its preclinical stage and at the onset of mild cognitive impairment (Figure 1). This feature also suggests that MCI is the same as AD in the biochemistry-related pathological mechanisms, in other words, mitochondria/oxidative stress hypothesis of AD applies mostly to, if not all cases, but at least the common, late-onset of AD. Hence, in the clinic, treatment of AD should be started at its very early stages, and mitochondria improvements should benefit the most AD patients. 

### 2.2. Antioxidant Defense System in Alzheimer's Dementia

Importantly, in mammalian cells, individuals have a powerful antioxidant system against free radicals/ROS, including small molecules and antioxidant enzymes. There is a defect in the antioxidant defense system, however, which may lead to oxidative damage in patients with AD. Glutathione (GSH) is the most abundant and important antioxidant in our bodies. Noninvasive magnetic resonance spectroscopy has shown that healthy young male and female subjects have higher amount of GSH in the parietal cortical region and a specific GSH distribution pattern (parietal cortex > frontal cortex > hippocampus ~ cerebellum), as well as higher amounts of GSH in the frontal cortical region compared to AD patients [47]. It has been found that erythrocyte antioxidant enzyme activities (catalase CAT, glutathione peroxidase GPX, and superoxide dismutase SOD) were significantly lower in patients with AD compared with controls [48]. Further more, glutathione reductase (GR) activity was lower in both MCI and AD patients than in aged subjects in relatively good health. Cognitive performance evaluated by the Mini-Mental State Examination (MMSE) was negatively associated with lipid peroxidation MDA levels (*r* = − 0.31, *P* = 0.028) and positively correlated with GR/GPX ratio in AD patients (*r* = 0.68, *P* < 0.001) [20]. These results suggest that alterations in these enzymes may play a role in the etiopathogenesis of AD. The expression of key oxidative stress-mitigating genes in different brain regions in AD has also been investigated using reverse transcriptase-polymerase chain reaction (RT-PCR) [49]. The levels of peroxidation mitigating (normalized CAT, GPX, and GR) mRNAs were elevated in the hippocampus and inferior parietal lobule, but not in the cerebellum of AD patients, which may reflect the protective gene response to the increased peroxidation in the brain regions showing severe AD pathology [49]. 

Decreased levels of polyunsaturated fatty acids and increased levels of markers of lipid peroxidation have been reported in the brain in AD, particularly in areas severely affected in the disease. 4-Hydroxynonenal (HNE), one marker of lipid peroxidation, is neurotoxic and elevated in AD brain and CSF. Furthermore, glutathione S-transferase (GST), a protective enzyme against aldehydes, and especially HNE, has been investigated in multiple brain regions in short-postmortem-interval AD patients [50]. A decrease in GST activity in all brain areas was observed in AD compared with age-matched controls and particularly in the amygdala, hippocampus, and parahippocampal gyrus. Expression of GST protein was also depleted in most regions of the brain in AD. These data indicate that reduced levels of the antioxidant enzyme GST have a role in the pathogenesis of neuron degeneration in AD [50].

Biliverdin reductase-A (BVR-A) is another antioxidant enzyme and drug target, together with heme oxygenase (HO); BVR-A forms a powerful system involved in the cell stress response during neurodegenerative disorders including AD. It has been shown that BVR-A undergoes posttranslational oxidative and nitrosative modifications in the hippocampus, but not the cerebellum, of subjects with AD and amnestic MCI [51, 52]. A significant increase of nitrated BVR-A was demonstrated only in AD and MCI hippocampi, whereas no significant modifications were found in cerebellar tissues. These results supported the hypothesis of a prevalence of nitrosative stress-induced modifications on BVR-A structure, and this evidence was confirmed by a significant upregulation of inducible nitric oxide synthase in hippocampal tissue of subjects with AD and MCI that was not present in cerebellum [51]. Therefore, nitrosative stress-induced modifications on hippocampal BVR-A are an early event in the pathogenesis of AD. In addition, HO-1 protein levels were significantly increased in the hippocampus of AD subjects, whereas HO-2 protein levels were significantly decreased in both AD and MCI hippocampi. Further, significant increases in Ser-residue phosphorylation together with increased oxidative posttranslational modifications were found in the hippocampus of AD subjects [53]. These data indicate that different forms of antioxidant enzymes may have different responses and roles to the oxidative stress in the brain. 

Oxidatively damaged molecules need to be removed or repaired. The ubiquitin-proteasome mechanism is involved in this process [54–56]. In Alzheimer's disease, there is a 2.5-fold increase in the total endosome volume in cortical neurons suggesting increased endocytic activity [57–59]. This increased rate of endocytosis in AD may be in response to the A*β* protein load or to the need to repair the damaged proteins in the brain, or both. It has been demonstrated that proteasomes are concerned with the removal of oxidized proteins, which plays an essential role in the cellular defense against oxidative attack [60].

To date, successful antioxidant therapy for AD has not been obtained. One reason is the difficulty of the most antioxidants to pass the blood-brain barrier. To overcome this problem, recently we and other research groups found that mitochondria-targeted catalase (MCAT) has a beneficial role in both transgenic AD model mice and wild-type mice through multiple mechanisms including reduction of ROS and oxidative damage, as well as toxic A*β*42 production and deposits in the brain [61, 62], further indicating that mitochondria and oxidative stress play an important role in AD and normal aging. 

Because increased oxidative damage and reduced antioxidant enzymes in the hippocampus and cortex are significantly involved in the development of AD (summarized in Figure 1); pathology-related local and global biomarkers in the individuals may help to detect the preclinical stages of disease and improve early and differential diagnosis. Core AD biomarkers, A*β* peptides (especially A*β*42 isoform) and phosphor-tau (p-tau) in cerebrospinal fluid (CSF), PET, and MRI are considered to be the most useful approaches in terms of diagnostic and predictive value in preclinical and clinical stages of AD [6, 63–68]. Importantly, a reduced concentration of A*β*1-42 in CSF may provide the earliest definitive evidence of Alzheimer's pathology in the brain since some individuals who have low concentrations of CSF A*β*1-42 show no evidence of amyloid on PET PIB scans. However, autopsy may show widespread diffuse—but minimal fibrillar—amyloid-plaque deposits [69]. Because CSF is the most cost-effective and reliable approach diagnostic, it is the focus of this paper.

### 2.3. Oxidative Stress in Dementia with Lewy Bodies

DLB has been defined clinically by the presence of dementia, gait/balance disorder, prominent hallucinations and delusions, sensitivity to traditional antipsychotics, and fluctuations in alertness [70]. These diagnostic guidelines have been confirmed by recent workshops [2, 71].

In pathology, DLB is one of the Lewy body diseases, so DLB shares some features of Parkinson's disease (PD). Filamentous protein inclusions in neurons (Lewy bodies, LB) and dystrophic neuritis (Lewy neuritis, LN) containing pathologic *α*-synuclein (*α*Syn) are the morphologic hallmarks of dementia with Lewy bodies and sporadic PD. They occur in the central, peripheral, and autonomic nervous system as essential or coincidental features. Their formation runs through several phases from initial dust-like particles cross-linked with *α*Syn to aggregation of ubiquitinated dense filaments, leading to the formation of LBs, in which *α*Syn is the major component and hundreds of other proteins may be included [72], to the final degradation and death of the afflicted neurons [8]. The disease progression-related decreases of dopamine, dopamine transporter (DAT), and tyrosine hydroxylase (TH) expression in striatum are negatively correlated with total substantia nigra (SN) *α*Syn burden and neuronal loss [4, 73]. Glutathione is the most abundant antioxidant involved. The reduced form (GSH(R)), but not oxidized glutathione (GSSG), has been shown to be dramatically altered in the SN of Lewy body disease patients post mortem; similarly, reduced but not oxidized glutathione levels in cerebrospinal fluid are lowered in Lewy body diseases therefore; it has been thought that the alterations of the glutathione system occur in a very early stage of the disease or may even represent a risk marker for LBD [74]. These pathological changes are important targets for disease diagnosis and therapy. For example, reduced DAT levels in DLB as shown with [123I]FP-CIT-SPECT is recognized as the most reliable and valid biomarker [75, 76].

Oxidative damage, particularly lipoxidation, advanced glycation (AGE), and AGE receptors (RAGE) in the cerebral cortex have also been seen in early stages of diseases with Lewy bodies [77]. Moreover, it demonstrates that *α*Syn lipoxidation is an early event in LBDs that precedes *α*Syn solubility modification, aggregation, and formation of Lewy bodies and neuritis [78]. It has been found that in human brain cortex, mitochondrial oxygen uptake, and complex I activity were significantly lower in PD and DLB, compared to healthy controls, whereas oxidative damage and mtNOS activity, cytochrome content, expression of Mn-SOD, and mitochondrial mass were significantly higher in the frontal cortex in PD and DLB [79]. The decreases in tissue and mitochondrial oxygen uptake and in complex I activity are considered the consequences of mitochondrial oxidative damage and mitochondrial dysfunction. The increases in mtNOS activity and in mitochondrial mass are interpreted as an adaptive response of the frontal cortex that involves increased NO signaling for mitochondrial biogenesis. The adaptive response would partially compensate for mitochondrial dysfunction in these neurodegenerative diseases and would afford a human evolutionary response to shortage of ATP in the frontal cortex. This compensatory mechanism also occurs in aged nonhuman primates [80].

Interestingly, shortened telomere length is associated with various age-related diseases, especially in dementia such as AD. Similar changes in telomere length in dementia with DLB have also been reported recently. Telomere length was significantly shorter in the DLB group than in the nondemented elderly control (NEC) group. Urinary 8-OHdG levels were significantly higher in the DLB group than in the NEC group. There was a negative correlation between telomere length and age in the DLB group [81]. 

The relationship between telomere length and oxidative stress has been investigated in several other diseases [82–85]. For example, telomere length was inversely correlated with oxidative stress in subjects with clinical depression [86]. Shorter leukocyte telomere length (LTL) is also associated with the presence of T2D and this could be partially attributed to the high oxidative stress in these patients [87]. In healthy women it has been shown that LTL is associated with cognitive performance [88], and decreasing telomere length was strongly correlated with decreasing hippocampal volume [89]. These preliminary data suggest that telomere length may be a possible early marker of DLB and other dementia risk. 

Heavy metals have been implicated as the causative agents for the pathogenesis of the most prevalent neurodegenerative diseases. Various mechanisms have been proposed to explain the toxic effects of metals ranging from metal-induced oxidation of protein to metal-induced changes in the protein conformation. Aggregation of *α*-synuclein is a key mechanism in Parkinson's disease and DLB, and various metals, including copper, constitute a prominent group of alpha-synuclein aggregation enhancers. Using a set of biophysical techniques, the *α*Syn-Cu21 binding sites were systematically characterized and analyzed for the possible role of metal binding in *α*Syn fibrillation. The analyses indicated that *α*Syn possesses at least two binding sites for Cu21. One of these binding sites is found in the N-terminal region [90].

 On the other hand, lipoxidative protein damage of aldolase A, enolase 1, and glyceraldehyde dehydrogenase (GAPDH) was found in the frontal cortex in the majority of cases of incidental Parkinson's disease (iPD), PD, and dementia with LB. Densitometric studies have shown that the ratio of oxidized protein per spot is higher in iPD, PD, and DLB compared with controls. These findings show oxidation of three enzymes linked with glycolysis and energy metabolism in the adult human brain as well as increased oxidation of aldolase A, enolase 1, and GAPDH in the frontal cortex in Lewy body diseases [91].

 In mammalian cells, glutathione peroxidase (GPX-1) is one of the main antioxidant enzymes inactivating hydrogen peroxide and protecting against oxidative stress. It has been found that the highest levels of GPX-1 occur in microglia with lower levels in neurons. Unstructured Lewy bodies were enveloped with a layer of GPX-1 that was partially colocalized with alpha-synuclein whereas concentric Lewy bodies had discrete deposits of GPX-1 around the periphery, which appeared to be involved in the degradation of the Lewy bodies. Abnormal alpha-synuclein as found in Lewy bodies produces hydrogen peroxide and these neurons are capable of directing antioxidant enzymes to regions of oxidative stress [92]. On the other hand, this endogenous defense may reflect a pathological mechanism and therapeutic target.

 Taken together, these findings support the idea that oxidative stress is involved in the pathological process of neurodegenerative diseases and antioxidant therapy in the treatment of DLB and AD is reasonable to reduce oxidative stress-associated neuronal damage [61, 93]. Further, oxidative stress-related biomarkers might have the potential to assist in the diagnosis of neurodegenerative diseases. 

## 3. CSF Biochemical Markers in AD and DLB

### 3.1. Core CSF Biomarkers and Oxidative Stress Markers in AD and MCI

Physiological processes and pathological changes in the brain can be monitored by analysis of cerebrospinal fluid (CSF). CSF markers reflect neuropathology in 94% of AD-associated cases of dementia [3]. As discussed above, A*β* depositions, neurofibrillary tangles, and oxidative damage are the main pathological features of AD. These feature-related biochemical changes in CSF are important signals for the disease initiation and development. For the past two decades, there has been intense interest in developing markers related to the neuropathology of AD in CSF. Several key CSF biomarkers of pathological processes in the brain are now available. CSF levels of 42-amino acid isoform of amyloid *β* (A*β*42), which directly induces reactive oxygen species, reflect brain amyloid pathology; levels of total tau reflect cortical axonal degeneration and levels of phospho-tau reflect tangle pathology [94]. While toxic A*β*42 forms and deposits plaques in the brain, the A*β*42 level in CSF is decreased accordingly. Most reports focused on these A*β* peptides and tau, showing mainly CSF A*β*42 is reduced but CSF A*β*40 and tau are increased in AD patients as well [1, 63].

Aging and amyloid *β* can induce oxidative stress and oxidative damage in the brain is considered the earliest event in AD. Accordingly, it is clear that CSF levels of A*β*42 retain diagnostic utility in patients with very mild AD and even mild cognitive impairment, and a combination of CSF t-tau and A*β*42 at baseline yielded a sensitivity of ~95% and a specificity of ~83% for detection of incipient AD in patients with MCI [95–98]. These data have been confirmed by a recent large-scale multicenter investigation [99–101]. It has been shown that cutoffs with sensitivity set to 85% were defined in the AD control groups and tested in the MCI group, where the combination of A*β*42/p-tau ratio and t-tau identified incipient AD with a sensitivity of 83% (95% CI, 78%–88%) and specificity of 72% (95% CI, 68%–76%) [100]. This identification of incipient AD with good accuracy, though less accurately than previous single-center studies, indicates a need for standardization of analytical techniques and clinical procedures, including the improvement of the analytical kits, for large-scale or multicenter studies [102–104]. Interestingly, cued recall deficits are most closely associated with CSF biomarkers indicative of AD (A*β*42/tau ratio, CSF AD+ group) in subjects with MCI [105, 106]. 

Though the diagnostic accuracies for AD decreased with age, the predictive values for a combination of biomarkers remained essentially stable, including the use of CSF biomarkers for AD, even in older populations [107]. The Alzheimer's Disease Neuroimaging Initiative (ADNI) has shown that diagnostic threshold CSF concentrations for A*β*42 and for the ratio t-tau/A*β*42 were determined in an ADNI-independent autopsy-confirmed AD cohort from whom antemortem CSF was obtained, and a clinically defined group of cognitively normal controls provides statistically significant separation of those who progressed from MCI to AD in the ADNI study. These data suggest that interrogation of antemortem CSF in cognitively impaired individuals to determine levels of t-tau, p-tau, and A*β*42, together with MRI and amyloid imaging biomarkers, could replace autopsy confirmation of AD plaque and tangle pathology as the “gold standard” for the diagnosis of definite AD in the near future [101].

Isoprostane is an important oxidative stress biomarker and it may be one of the earliest markers for neuronal damage related to AD [108]. It has been shown that cerebrospinal fluid lipoproteins are more vulnerable to oxidation in AD and cerebrospinal fluid levels of F2-IsoPs are elevated in probable AD patients compared to age-matched controls [109–111]. Furthermore, combined analysis of CSF Ab42 and F2-IsoP levels largely preserved sensitivity and improved specificity relative to classification with A*β*42 and tau alone, indicating its utility as an AD biomarker [112]. Analysis of CSF F2- IsoPs in 421 clinically normal individuals has shown a significant increase in CSF F2- IsoPs over the adult human lifespan (*P* < 0.001) [113], indicating a correlative relationship between free radical injury in the central nervous system and aging. In addition, increased CSF F2-IsoPs levels were present in clinically normal subjects with the biomarker signature of AD (*P* < 0.05) and those subjects with increased CSF tau (*P* < 0.001) [113]. Elevated F2-IsoP levels in the CSF were also found in preclinical familial AD (FAD) mutation carriers [114]. Interestingly, isoprostane may be a predictive parameter for the development of AD. Cognitively normal (NL) individuals with a maternal history (MH) of late-onset Alzheimer's disease (LOAD) showed higher IsoP and reduced A*β*(42/40) CSF levels compared with NL with a paternal history (PH) and NL with a negative family history of LOAD (NH) (*P* values ≤.05), whereas no differences were found between NH and PH. No group differences were found for p-tau(231) and t-tau. The IsoP and A*β*(42/40) levels were correlated only within the MH group (*R*² = .32, *P* = .005). Therefore, adult children of LOAD-affected mothers express a pathobiological phenotype characterized by A*β*-associated oxidative stress consistent with AD, which might reflect increased risk for developing the disease [115] (Table 1). 

Very interestingly, some markers of oxidative damage are also elevated in mitochondria isolated from AD lymphocytes. This not only provides insight into the important role of oxidative stress and mitochondria in AD development but also indicates that these oxidative stress indices potentially could serve as a viable biomarker for this dementing disorder [22]. 

A novel misfolded protein assay for the detection of soluble oligomers composed of A*β* x-40 and x-42 peptides from CSF has also been developed. Preliminary validation of this assay with 36 clinical samples demonstrated the presence of aggregated A*β*x-40 in the CSF of AD patients. Together with measurements of total A*β*42, diagnostic sensitivity and specificity greater than 95% and 90%, respectively, were achieved. Although larger sample populations will be needed to confirm this diagnostic sensitivity, these studies demonstrate a sensitive method of detecting circulating A*β*x-40 oligomers from AD CSF and suggest that these oligomers could be a powerful new biomarker for the early detection of AD [116].

Mitochondrial coenzyme Q-10 (CoQ) is an antioxidant that receives electrons from complex I and II. It has been shown that the percentage of oxidized/total CoQ (%CoQ) in the CSF of the AD group (78.2 ± 18.8%) was significantly higher than in the control group (41.3 ± 10.4%) (*P* < 0.0001). The concentration of 8-OHdG in the CSF of AD patients was greater than in the CSF of controls (*P* < 0.0001) and was positively correlated with the duration of illness (*r*(*s*) = 0.95, *P* < 0.0001). The %CoQ was correlated with concentrations of 8-OHdG in the CSF of AD patients (*r*(*s*) = 0.66, *P* < 0.001), further suggesting both mitochondrial oxidative damage and oxidative DNA damage play important roles in the pathogenesis of early AD development, and CoQ may be a useful biomarker for the disease [117].

As described above, there is a significant decrease in antioxidant GST activity and protein expression in multiple brain regions, especially the hippocampus in short-postmortem-interval AD patients [50]. Similarly, a significant decrease in GST activity and protein levels was also found in ventricular CSF in AD [50], indicating that reduced levels of antioxidant enzyme GST may have an important role in the pathogenesis of AD and may serve as a CSF biomarker for this degenerative disease.

Overall, the primary focus of AD-associated study has been directed toward amyloid and tau pathology and their relations to synaptic and neuronal loss. However, as the complexity of the disease becomes increasingly evident, the importance of other factors such as oxidative stress and mitochondrial dysfunction becomes apparent. Therefore, in addition to the core biomarkers (reduced A*β*42 and increased tau in AD CSF), oxidative stress-related biomarkers may also have potential utility in diagnosis of AD, including preclinical and very early-stage disease. The principal AD markers are listed in Table 1. 

### 3.2. Oxidative CSF Biomarkers in DLB

The clinical diagnosis of dementia with Lewy bodies (DLB) is made on the basis of consensus criteria; however, the sensitivity of the criteria is relatively low [9]. Differentiating dementia with Lewy bodies from AD can be difficult because of the substantial overlap in clinical features. A classification of patients with AD and patients with other dementias accomplished by combination of CSF A*β*42 and p-tau shows that the CSF AD biomarker profile was seen in 47% of patients with DLB [3]. However, from many reports, a conclusive proposal, that is, a combination of CSF measures appears to emerge, that may well be able to differentiate DLB from other dementias: *α*-synuclein reduction in early DLB, a correlation between CSF *α*-synuclein and A*β*42 measures (characteristic for DLB only), and t-tau and p-tau181 profile (differentiating AD from DLB) [9].

Furthermore, using the quantitative A*β*-immunoblot to analyze CSF samples of neuropathologically defined patients with AD (definite AD, dAD) and DLB (definite, dDLB), the authors found that A*β* (1–42%) was significantly lowered in dAD compared to nondemented controls (NDC) (*P* = 1.6 × 10^−7^). Oxidized A*β* (1–40ox%) was elevated in dDLB as compared to NDC (*P* = 1.8 × 10^−5^). Thus, it confirmed previous results on A*β* peptide patterns in neuropathologically characterized patients with AD and DLB (Table 1). The results underscore the usefulness of CSF A*β*(1–42%) and A*β*(1–40ox%) as diagnostic biomarkers for AD and DLB, respectively [118, 119]. As mentioned above, reduced GSH but not oxidized GSSH in CSF was significantly lower in Lewy body disease patients than control subjects [74]. To date, the implications of reduced GSH observed in AD patients are not yet clear (Table 1). However, differences in total glutathione concentrations in CSF for several neurodegenerative diseases including AD were not significant between groups [120], indicating that unlike reduced glutathione, the total glutathione is not useful as a CSF marker for assumed oxidative stress in patients with PD, AD, or multiple system atrophy.

Altered metal homeostasis may play a role in the pathogenesis of neurodegenerative disorders. It has been shown that patients with DLB had elevated Ca and Mg levels in CSF and Mg levels in plasma when compared to all other groups including AD and healthy controls (*P* < 0.001). A combination of CSF-Mg and CSF-Ca could distinguish DLB from AD with a sensitivity of 93% and a specificity of 85% [121]. Cu levels in both CSF and plasma tended to be higher in DLB compared to the other groups, but these trends failed to reach significance after correction for multiple comparisons. The observed elevations of CSF-Mg, CSF-Ca, and CSF-Cu may contribute to, or be associated with, the neurodegenerative process in DLB. Therefore, CSF-Mg and CSF-Ca may be a valuable tool in distinguishing DLB from AD.

Neprilysin (NEP) is an A*β*-degrading protein found at presynaptic terminals and in body fluids. Reduced CSF NEP activity levels have been shown to occur in early AD; similarly, demented Lewy body disease patients had lowered CSF NEP activity levels, compared to both non-demented Lewy body disease subjects (*P* = 0.004) and controls (*P* = 0.02). In addition, CSF NEP activity levels correlated positively with CSF A*β*42 levels which was not explained by the presence or absence of ApoE4 [122], suggesting that NEP is involved in both AD and DLB and altered CSF NEP activity may be a useful biomarker for dementia. Further, cystatin C (CysC) is a carrier of soluble A*β*42 in CSF and reduces A*β* plaque formation. Demented Lewy body disease patients had decreased CSF CysC levels. The correlation between CSF CysC and A*β*42 levels was high in non-demented subjects, but poor in demented patients, indicating that low CSF CysC levels are associated with dementia, possibly through a disturbed elimination of soluble A*β*42 [123].

Hyaluronic acid (HA) is an adhesion molecule known to regulate both vascular and inflammatory processes. Recent analyses showed that male AD and DLB patients had almost double the amount of HA compared to female patients whereas no gender differences were observed in the controls. Furthermore, CSF levels of HA in most female AD patients correlated with various AD-related biomarkers. Correlations between HA levels and markers of inflammation and vascular changes were only detected in female AD patients but in both male and female DLB patients. Therefore the HA profile in CSF, but not in plasma, and associations to other markers appear to be gender dependent which should be taken into account in clinical examinations and future biomarker studies [124].

Some hopeful DLB biomarkers in CSF are listed in Table 1, including neuropeptide CART discussed below. 

## 4. Neurotransmitter CART, a Potential Biomarker for DLB or Causative Factor for Dementia

CART peptide is broadly expressed in the cortex, hippocampus, amygadala, hypothalamus, pituitary, and some endocrine glands [125, 126]. This suggests a general role in different cells. Very recently, we found that CART has a general cytoprotection function in mammalian and human cells [11]. We found that CART protects mitochondrial DNA (mtDNA), cellular proteins, and lipids against the oxidative action of hydrogen peroxide. Using cis-parinaric acid as a sensitive reporting probe for peroxidation in membranes and a lipid-soluble azo initiator of peroxyl radicals, we found that CART has an antioxidant property. CART is preferentially localized in mitochondria, which are the main source of free radicals. Interestingly, this antioxidant and protective role is also seen in a mouse PD model [11]. Therefore we propose that CART is an important antioxidant hormone. These mitochondria associated cytoprotection and trophic roles are particularly important in the development of neurodegenerative diseases, including AD and DLB, since most of them have a mitochondrial dysfunction or an energy failure condition. 

MRI study has shown that patients with DLB displayed hypothalamic atrophy whereas this region was not affected in AD patients [127]. CART is a neuropeptide expressed selectively in neurons in the hypothalamus. It is, therefore, possible that neurons producing CART in the hypothalamus are affected in DLB. Although Lewy bodies have been described in the hypothalamus of DLB patients [128], extensive neuropathological studies of this region in DLB have not yet been reported. Consequently, it would be of interest to study whether neurons expressing CART are affected in DLB as well as analyzing the role of changed CSF-CART levels. Using sensitive and commercially available radioimmunoassay (RIA), Schultz et al. [129] found that CSF-CART levels were significantly reduced by 30% in DLB patients compared to controls as well as AD patients. These results suggest that reduced CSF CART is a sign of hypothalamic dysfunction in DLB and that it may serve as a new biomarker for DLB patients. A summary of CSF biomarkers for DLB is shown in Table 1. 

Interestingly, CSF dopamine metabolite (DOPAC) level is correlated with behavioral and psychological signs and symptoms of dementia (BPSD) in general [130]. Intracerebroventricular injections of CART in rodents lead to increased dopaminergic activity in nucleus accumbens, striatum, and hypothalamus [131] and systemic injections of CART in a PD mouse model preserve dopaminergic neurons in SN area [11]. Reduced CART levels may, therefore, be involved in causing or augmenting the dopaminergic hypofunction resulting in Parkinsonism as well as other symptoms, including mood/psychiatric symptoms [132]. CART level is also reduced in AD patients [129]. The precise mechanisms of CART underlining these changes and the significance of CART in both DLB and AD diseases need further investigation in the future. 

Although the involvement of CART in mitochondria and neurotrophin signaling in healthy and injured neurons is increasingly recognized, the impact of CART on the cellular processing of amyloid precursor protein tau and alpha-synuclein, the key genes/proteins associated AD and DLB, is currently unexplored.

## 5. The Differences between Central and Peripheral Biomarkers

The utility of plasma A*β*42 and other A*β* peptides as biomarkers for AD is less compelling and concentrations of A*β*42 in cerebrospinal fluid and plasma show no apparent correlation [69, 133–138]. However, since the blood test is noninvasive, easily applicable, and cost effective, peripheral biomarkers especially blood or serum biomarkers are definitely desirable, and some reports suggest that they may be promising biomarkers. 

Alzheimer's dementia is a systemic or global metabolism disorder. Some important changes in the body or circulatory system may occur much earlier than clinical onset. Plasma A*β* levels are elevated in early-onset AD caused by autosomal dominant mutations. Notably, it has been reported that plasma A*β*42, but not A*β*40, is also significantly elevated in late-onset AD first degree, compared to controls, determined by using a specific monoclonal antibody against A*β*42 or A*β*40 [139]. Similarly, higher plasma A*β*42 at baseline was a significant predictor for the conversion to probable or possible AD at 5 years. Higher conversion to AD was also associated with male gender but not with either higher scores on the Geriatric Depression Scale, stroke, cerebral infarction, or apolipoprotein E *ε*4 allele [140]. This A*β*42 level may be changed in the progression of the disease prior to the development of overt dementia, due to the formation of A*β* plaques in the brain [114]. 

In contrast to CSF, plasma and urinary F2-IsoPs are not increased in probable AD patients [141]. In combination, these data indicate that CSF F2-IsoPs, but not peripheral F2-IsoPs, are biomarkers of oxidative damage to the brain in AD. Since arachidonic acid is distributed among neuronal and nonneuronal elements in brain, F2-IsoPs are not specific for neuronal oxidative damage; they reflect a total oxidative damage (lipid peroxidation) in the brain [112, 142]. 

The AD brain is marked by severe neuronal death, which has been partly attributed to increased oxidative stress. The pathophysiology accounting for this free radical injury is not well delineated at this point but it is thought that a derangement in transition metal metabolism contributes to the process. Significantly decreased loosely bound iron in the hippocampal white matter of mild-moderate and severe AD patients and a trend towards increased nonheme iron in the hippocampal gray matter of severe AD patients have been observed. Furthermore, decreased levels of total copper were seen in severe AD and DLB frontal cortex compared to controls, suggesting an imbalance in brain metal levels in both AD and DLB. The decrease in loosely bound iron in mild-moderate AD patients may be associated with myelin breakdown seen in the beginning stages of AD and implies that iron dysregulation is an early event in AD pathogenesis [143]. 

Interestingly, peripheral derangement of transition metal metabolism is present early in the dementing process. An increase in the ratio of serum copper to nonheme iron levels predicted which subjects with mild cognitive impairment would progress to dementia versus those who would remain cognitively stable [144]. No gene was identified as being dysregulated more than 2-fold in a cDNA-based microarray (IronChip) containing genes relevant to iron and copper metabolism used to assess transition metal metabolism in circulating lymphocytes from cognitively normal and demented subjects. Therefore, the increased ratio of serum copper/iron prior to dementia has potential as a biomarker for cognitive decline and mirrors other changes in serum previously reported by others, but iron and copper metabolism pathways appear to be broadly unaffected in peripheral blood in AD [144].

 Oxidative stress in dementia is not only a brain condition but also a global problem, and it may be monitored by urinary markers. 8-Hydroxy-2′-deoxyguanosine (8-OHdG) is a biomarker indicating oxidative DNA damage. Paraoxonase 1 (PON1) is a high-density lipoprotein (HDL)-associated antioxidant enzyme and prevents oxidation of low-density lipoproteins. Urinary 8-OHdG levels were found to be significantly increased, but serum PON1 activity was significantly decreased in AD patients compared to controls [145]. Hence, oxidant stress and oxidative DNA damage are important pathological processes in AD, and such oxidation related status of patients with AD might be easily determined and monitored by these biomarkers, urinary 8-OHdG level and serum PON1 activity. These peripheral biomarkers totally reflect an imbalance of oxidant and antioxidant and the systemic condition of oxidative stress, supporting the view that mitochondrial dysfunction and oxidative stress may be the initial events prior to the amyloid accumulation (Figure 1). This mitochondria/oxidative stress hypothesis may apply mostly to the late onset of AD [146, 147].

 Some neurotrophic factors in serum may be changed in dementia patients. Glial cell-line derived neurotrophic factor (GDNF) has been extensively studied for its neuroprotective role in vitro and in vivo. Straten et al. compared GDNF concentrations in CSF and serum of patients with AD and normal controls (NC). While GDNF concentrations in CSF were significantly increased in patients with AD (291.7 pg/mL) compared with NC subjects (218.7 pg/mL, *P* = 0.012), GDNF concentrations of AD patients (486.5 pg/mL) in serum were significantly decreased compared with the NC group (711.5 pg/mL, *P* < 0.001). It was thought that increased GDNF in CSF of AD might be due to an upregulated expression in the CNS as an adaptive process of the impaired brain to enhance neurotrophic support at least in early stages of disease and/or impairment of CSF turnover [148]. It is curious that CSF GDNF increased but serum GDNF significantly decreased in AD patients.

 A general and unbiased approach to the identification of diagnostically useful antibodies that avoids the requirement for antigen identification has been developed recently [149]. This method involves the comparative screening of combinatorial libraries of unnatural synthetic molecules against serum samples obtained from cases and controls. Molecules that retain far more IgG antibodies from the case samples than the controls are identified and subsequently tested as capture agents for diagnostically useful antibodies. Using this method, two candidate IgG biomarkers for AD have been identified; they are AD peptoids: ADP1- (or 3-) binding antibodies and ADP2-binding antibodies. AD blood samples contained higher levels of antibodies that bind to ADP2 as well as antibodies that bind to ADP1 and ADP3 than non-AD samples [149], indicating they may be strong biomarkers for the disease. Certainly, more research is needed to determine whether the peptoids ADP1–3 will be useful reagents for the clinical diagnosis of AD.

Taken together, oxidative stress in dementia is probably a global pathological feature, it can be monitored by some central and peripheral biomarkers. A*β* biomarkers are likely downstream markers of mitochondrial and metabolism dysfunction [146]. Compared to CSF biomarkers for dementia, peripheral biomarkers are relatively less accurate and less consistent, probably because dementia is mainly a brain disorder and the local pathological factors closely reflect the nature of the disease. Array-based methods and other sensitive approaches may provide new, useful biomarkers for quick and accurate diagnosis of dementia and more readily distinguish its subtypes.

## 6. Conclusions

Oxidative stress is significantly involved in the pathology of degenerative Alzheimer's dementia and dementia with Lewy bodies, even in the early stages of the diseases. This feature supports an oxidative stress hypothesis for dementia (Figure 1). Disease-related biomarkers (i.e., oxidative stress-related chemicals) can provide diagnostic, prognostic, and therapeutic targets and also may address some etiological questions and important problems in clinical and translational medical research. 

Importantly, not only in classic AD patients but also in people with mild cognitive impairment, CSF A*β*42 levels are significantly decreased when compared to healthy adults. This reflects abnormal amyloid metabolism, one of the most important changes in the brain, suggesting the formation of some tiny amyloid plaques in the hippocampus and the cortex. At the same time or later, total tau levels, particularly p-tau levels, are largely increased in AD patients comparing to healthy controls.

Patients with DLB show either normal levels of these core CSF biomarkers or slightly elevated T-tau and slightly decreased A*β*42, while P-tau levels are around normal or only slightly elevated or slightly decreased. This distinguished DLB from AD and can be the first key point of the differential diagnosis. Furthermore, *α*Syn is decreased in most DLB cases compared to healthy controls as well as AD patients. This is the second distinction that may assist in the differential diagnosis. 

In addition, among other biomarker candidates, endogenous antioxidant CART is very promising since deficits in serotonergic and dopaminergic pathways seem more pronounced in DLB patients, and CART can modulate these pathways [132]. In particular, clinic practice shows CART level in CSF is significantly decreased in DLB patients compared to AD as well as healthy controls [129]. Combining CART with the core markers may further increase diagnostic accuracy (Table 1). 

In conclusion, the combination of CART and the brain specific proteins t-tau, p-tau, A*β*42, and *α*Syn in CSF are associated with the clinical diagnosis of DLB and discriminate between AD and DLB with high diagnostic accuracy, suggesting this combination as a new potential biomarker panel for DLB. If this diagnosis panel is confirmed by new investigations, it may be a great advance in neurodegenerative disease diagnostics and treatment.

## Figures and Tables

**Table 1:** Differences of main CSF biomarkers in DLB and AD.

	A*β*42	T-tau	P-tau	*α*Syn	CART	A*β*40ox	F_2_-IsoP	GSH
Healthy	—	—	—	—	—	—	—	—
DLB	— or ↓	—	—	↓	↓↓	↑	?	↓
AD	↓↓	↑↑	↑↑	—	↓	—	↑	?

—: normal level; ↓: decrease; ↑: increase; double arrows: full changes; ?: unknown.
